# Modeling of a tissue expander with a radiofrequency identification port in postmastectomy radiation therapy planning

**DOI:** 10.1093/jrr/rrae004

**Published:** 2024-03-08

**Authors:** Fumiyasu Matsubayashi, Taro Takahashi, Hikaru Miyauchi, Yasushi Ito, Arisa Harada, Yasuo Yoshioka

**Affiliations:** Radiation Oncology Department, Cancer Institute Hospital, Japanese Foundation for Cancer Research, 3-8-31 Ariake, Koto-ku, Tokyo 135-8550, Japan; Radiation Oncology Department, Cancer Institute Hospital, Japanese Foundation for Cancer Research, 3-8-31 Ariake, Koto-ku, Tokyo 135-8550, Japan; Radiation Oncology Department, Cancer Institute Hospital, Japanese Foundation for Cancer Research, 3-8-31 Ariake, Koto-ku, Tokyo 135-8550, Japan; Radiation Oncology Department, Cancer Institute Hospital, Japanese Foundation for Cancer Research, 3-8-31 Ariake, Koto-ku, Tokyo 135-8550, Japan; Radiation Oncology Department, Cancer Institute Hospital, Japanese Foundation for Cancer Research, 3-8-31 Ariake, Koto-ku, Tokyo 135-8550, Japan; Radiation Oncology Department, Cancer Institute Hospital, Japanese Foundation for Cancer Research, 3-8-31 Ariake, Koto-ku, Tokyo 135-8550, Japan

**Keywords:** postmastectomy radiation therapy, tissue expander, radiofrequency identification, modeling

## Abstract

The purpose of this study was to evaluate the dose attenuation of Motiva Flora® (Flora, Establishment Labs, Alajuela, Costa Rica) tissue expander with a radiofrequency identification port locator and to develop a model for accurate postmastectomy radiation therapy planning. Dose attenuation was measured using an EBT3 film (Ashland, Bridgewater, NJ), and the optimal material and density assignment for the radiofrequency identification coil for dose calculation were investigated using the AcurosXB algorithm on the Eclipse (Varian Medical Systems, Palo Alto, CA) treatment planning system. Additionally, we performed *in vivo* dosimetry analysis using irradiation tangential to the Flora tissue expander to validate the modeling accuracy. Dose attenuations downstream of the Flora radiofrequency identification coil was 1.29% for a 6 MV X-ray and 0.99% for a 10 MV X-ray when the coil was placed perpendicular to the beam. The most suitable assignments for the material and density of the radiofrequency identification coil were aluminum and 2.27 g/cm^3^, respectively, even though the coil was actually made of copper. Gamma analysis of *in vivo* dosimetry with criteria of 3% and 2 mm did not fail in the coil region. Therefore, we conclude that the model is reasonable for clinical use.

## INTRODUCTION

Breast reconstruction after mastectomy is a cosmetic option for patients who have undergone breast cancer surgery. It has many benefits for patients, including improved body image and quality of life [1]. In many cases, two-stage breast reconstruction is involved, in which a tissue expander (TE) is temporarily inserted into the chest wall and later replaced with a permanent implant. The TE has a saline injection port, needle guide and a port locator. The port locator, often using magnets, helps identify the injection port’s location by magnetic detection from outside the body.

Postmastectomy radiation therapy (PMRT) can reduce local and regional lymph node recurrence rates and improve survival [2, 3]. However, for 6 MV X-ray PMRT in the presence of a TE, it is reported that a 22% dose attenuation occurs downstream of the magnet [4]. For dose changes in such heterogeneous regions, documentation published by the European Society for Radiotherapy and Oncology (ESTRO booklet No. 7) [5] recommends an accuracy within 3% for inhomogeneity areas in treatment planning. The dose attenuation can be effectively considered by modeling the magnet in the radiotherapy planning system (RTPS) [6, 7].

Recently, Motiva Flora® (Flora, Establishment Labs, Alajuela, Costa Rica), a TE with a radiofrequency identification (RFID) port locator and plastic needle guide, was developed. This magnet-free port locator has the advantage of allowing magnetic resonance imaging with the TE inserted [8]. For PMRT in the presence of Flora TE, Hwang *et al*. [9] examined the acceptability of dose variation by comparing dose distributions with magnet-conducted TE. However, dose attenuation needs to be evaluated by measurement because the RFID coil is made of copper, which can affect accuracy of treatment planning. More specifically, to perform dose calculations, the AcurosXB algorithm in the Eclipse (Varian Medical Systems, Palo Alto, CA) requires the assignment of materials and densities to high computed tomography (CT)-value regions. Therefore, appropriate materials and densities need to be assigned to the RFID coil to ensure accurate planning. The purpose of this study was to evaluate the dose attenuation of the Flora TE RFID coil and to develop a model for accurate PMRT planning using the AcurosXB algorithm.

## MATERIALS AND METHODS

### Port structure of the Flora TE

**Fig. 1:**
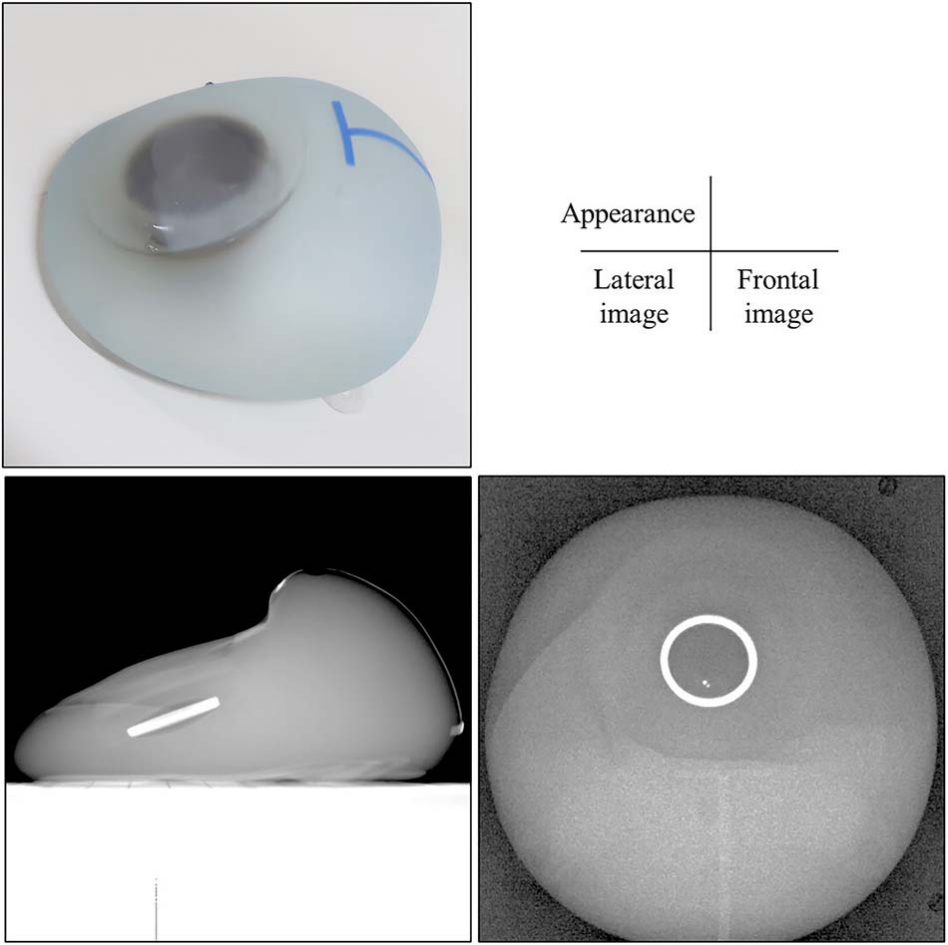
The appearance and X-ray images of Flora TE.

Figure 1 shows the appearance and X-ray images of the Flora TE. Most of the parts in the port are made of Polyetheretherketone (PEEK) resin. The RFID coil is located in the PEEK resin and is made of copper (density: 8.96 g/cm^3^). It has a circular diameter of 24.7 mm, a thickness of 2 mm and a height of 2.1 mm [9].

### Measurement of dose attenuation by the Flora TE RFID coil

**Fig. 2:**
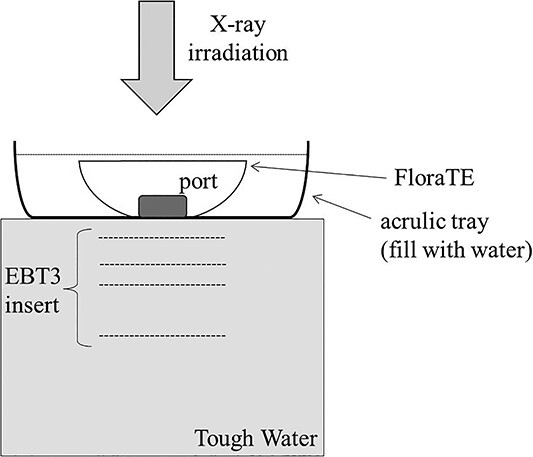
Schematic illustration of the dose attenuation measurement.

Figure 2 shows a schematic illustration of the measurement of dose attenuation. We placed Tough Water (Kyoto Kagaku, Kyoto, Japan) phantom on the treatment couch to make it 20 cm thick, and placed an acrylic tray on it. We then placed the Flora TE, which was filled with saline, in the tray with the port facing down, and filled the tray with water to a height of 6 cm. EBT3 (Ashland, Bridgewater, NJ) films were inserted into the Tough Water phantom parallel to the port face at depths of 0, 1, 3, 5 and 10 cm from the surface of the phantom. The Flora TE was irradiated with X-ray beams from TrueBeam (Varian Medical Systems), and the transmitted dose was measured by EBT3. Table 1 summarizes the settings used for the irradiations. We converted the optical density measured by the EBT3 to dose using a calibration table acquired by irradiating a Tough Water phantom.

**Table 1:** Summary of the irradiation settings

**Settings**	**Values**
Energy	6 MV and 10 MV
MU	250 MU
Field size	10 cm^2^ at Tough Water surface
Source to surface distance	100 cm at Tough Water surface

### CT acquisition and treatment planning for the modeling

For the modeling, we acquired CT images of the Tough Water phantom and the Flora TE using the same settings as used for the attenuation measurement. The CT images were acquired using Aquilion ONE (Canon Medical Systems, Otawara, Japan), which has an upper CT-value limit of 29 768 Hounsfield unit (HU). To reduce metal artifacts from the coil, a metal artifact reduction technique (SEMAR) was applied to the CT images. Prior to this study, we verified the constancy of CT-value and the absence of image distortion in applying SEMAR following the American association of physicists in medicine task group No. 66 report [10]. The other scan and reconstruction parameters are summarized in Table 2. These parameters, except for the scan mode which was modified to address water surface blurring with the CT couch movement, matched clinical use.

**Table 2:** Summary of scan parameters

**Parameters**	**Values**
Tube voltage	120 kV
Tube current	600 mA
Scan mode	Volume (nonhelical)
Rotation speed	1 s
Field of view	50 cm
Matrix size	512 × 512
Slice thickness	2 mm

Treatment planning was performed using Eclipse ver. 16.1. After importing the CT images into the Eclipse, we contoured the coil using ‘Segment High Density Artifacts’ function, which automatically detected regions of high CT-value to which materials and density values should be assigned. Treatment planning was performed with the same setting as the attenuation measurement, and doses were calculated using the AcurosXB algorithm with the 2 mm calculation grid, reflecting clinical practice.

### Modeling procedure

The optimal assignment of material and density to the coil in the treatment planning was investigated by comparing the measured dose profile with the calculated dose profile. We selected three materials for consideration as the most appropriate: aluminum, titanium and stainless steel. In the AcurosXB algorithm, there is a limit to the density that can be assigned to each material, so the densities for each material were assigned values closest to the density calculated from the CT-values of the coil using an HU–density conversion table. Note that our facility has registered aluminum, titanium and stainless steel as non-biological materials in the conversion table. For the optimal density investigation, three aluminum densities were evaluated: 2.70 g/cm^3^ that is the actual physical density, and the minimum and maximum values within the provided range (2.27 g/cm^3^ and 3.56 g/cm^3^, respectively).

The dose profiles were compared using the Simple IMRT analysis (Triangle products, Kashiwa, Japan). All the EBT3 films imported into the Simple IMRT analysis were processed with a 5 × 5 pixel median filter. Previous studies reported a 1.5% uncertainty in film dosimetry due to nonuniformity [11]. To address the nonuniformity, we averaged each film with its flipped counterpart. The dose difference (DD) between the calculated dose and the measured dose within a 3 cm square centered on the coil was calculated for all depths. Next, the pass rates were calculated when the DD criteria were set to 1%. The average pass rates for all depths were then determined, and the materials and densities with the best average pass rates were investigated.

### Validation of the modeling

**Fig. 3:**
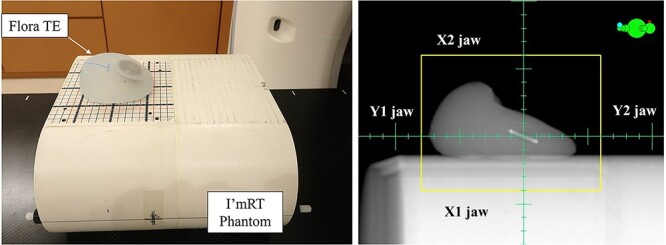
Flora TE and the phantom placement in the modeling validation procedure and the digitally reconstructed radiography. Left side: flora TE and the phantom placement, Right side: the digitally reconstructed radiography of the Flora TE.

We created a tangential irradiation plan for the Flora TE and evaluated the validity of the modeling using *in vivo* dosimetry. In phantom preparation for this, the Flora TE was placed on the I’mRT Phantom (IBA dosimetry, Schwarzenbruck, Germany) with the port facing up (Fig. 3). CT images were acquired using Aquilion ONE. The scan parameters were identical to those in Table 2, with the exception of the scan mode, which was helical scan in this investigation to align with clinical use. The coil was detected using the ‘Segment High Density Artifacts’ function in the Eclipse, and material and density were assigned according to the value that resulted in the best average pass rate in the modeling study. In treatment planning, the irradiation parameters were set with the values shown in Table 3. Digitally reconstructed radiography of this validation plan is shown in Fig. 3. Dose calculation was performed using the AcurosXB algorithm.

**Table 3:** Parameter setting in validation plan

**Settings**	**Values**
Energy	6 MV and 10 MV
MU	250 MU
Gantry angle	271° (parallel to the coil)
Collimator angle	0°
Isocenter	Center of the coil
Field size	X: 10 cm, Y: 14 cm (asymmetry)
Multi leaf collimator	None

The tangential irradiations to the Flora TE were performed using the TrueBeam, and transmitted beam images were acquired using an electronic portal imaging device (as1200, Varian Medical Systems). In the irradiation, source to imager distance was set at 150 cm (pixel size in the imager plane was 0.336 mm). The images were transferred to SunCHECK patient (SunNuclear, Melbourne, USA) software platform for *in vivo* dose analysis. In the analysis, we calculated gamma pass rates with a 3% of dose deviation to the maximum dose and 2 mm of distance to agreement.

## RESULTS

### CT images of the port

**Fig. 4:**
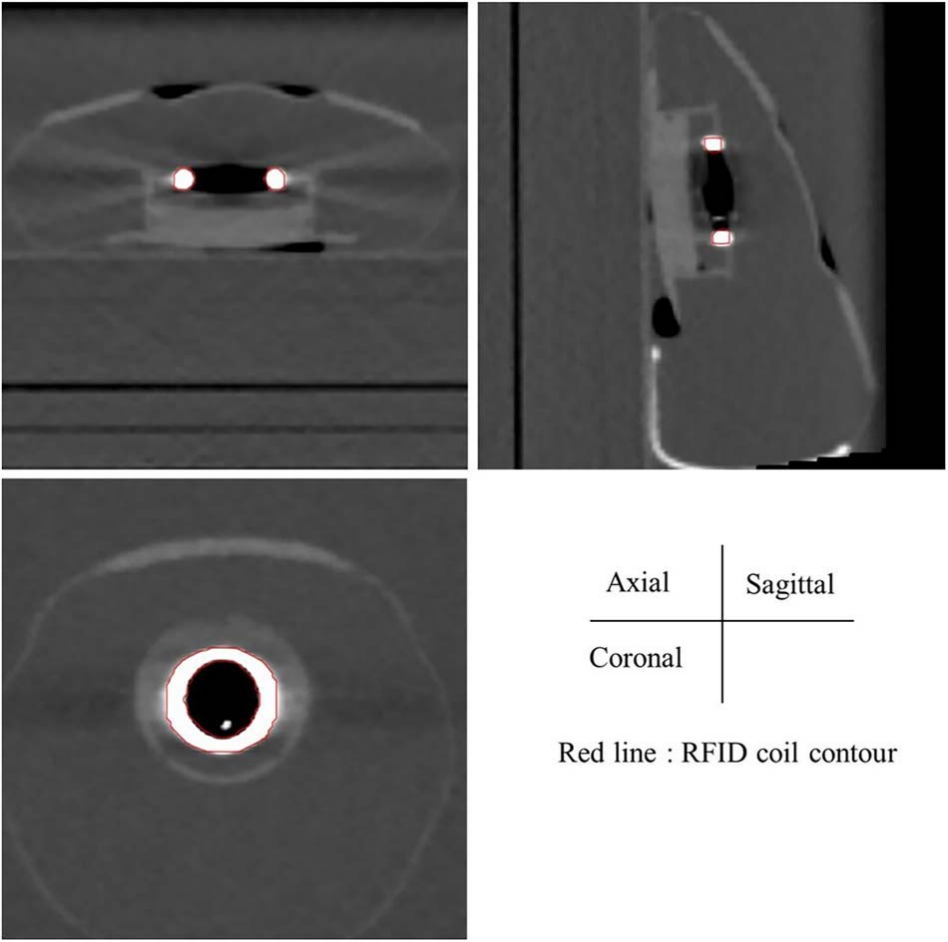
CT image acquired in the modeling study. Lines indicate the coil contour drawn by the ‘Segment High Density Artifacts’ function.

The CT image acquired in the modeling study and the contour of the coil are shown in Fig. 4. Despite metal artifacts present near the coil, its shape remained identifiable. An internal air cavity was also visible within the coil. The mean CT-value within the contour was 3771 HU, with a maximum of 17 000 HU and a standard deviation of 5660 HU. The density calculated using the conversion table based on the mean CT-value was 2.75 g/cm^3^.

### Dose profile comparison and the modeling

**Fig. 5:**
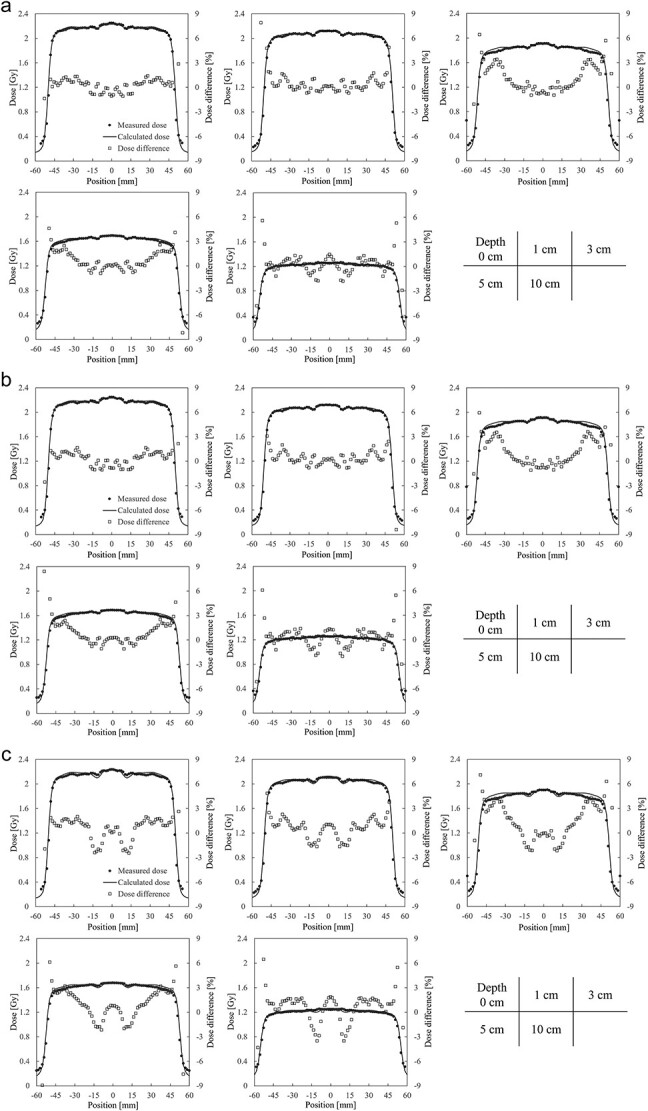
(**a**) Measured and calculated dose profiles for 6 MV X-ray with aluminum assignment. (**b**) Measured and calculated dose profiles for 6 MV X-ray with titanium assignment. (**c**) Measured and calculated dose profiles for 6 MV X-ray with stainless steel assignment.

**Fig. 6:**
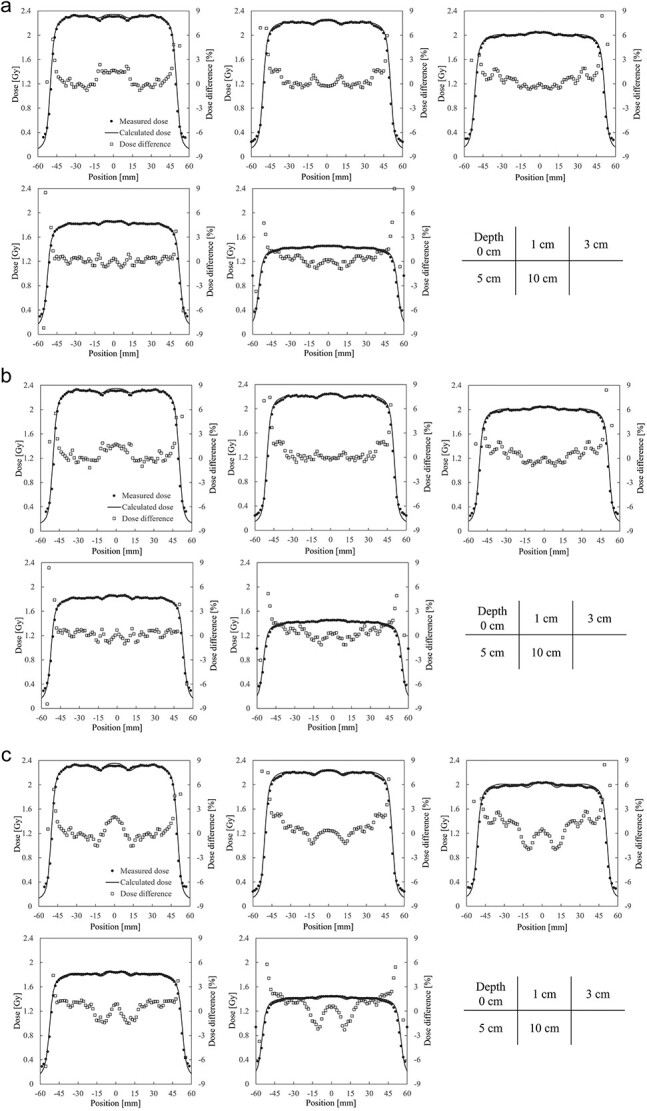
(**a**) Measured and calculated dose profiles for 10 MV X-ray with aluminum assignment. (**b**) Measured and calculated dose profiles for 10 MV X-ray with titanium assignment. (**c**) Measured and calculated dose profiles for 10 MV X-ray with stainless steel assignment.

**Fig. 7:**
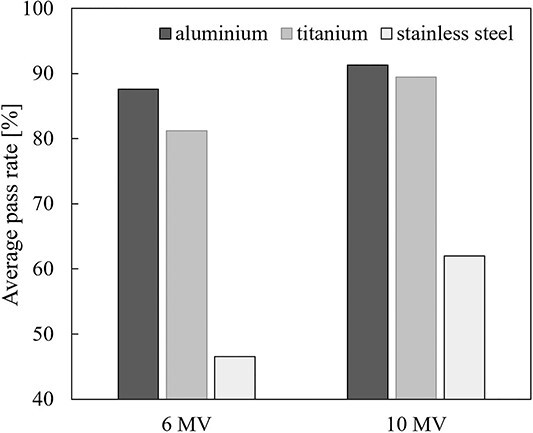
The average pass rates of each material.

Figures 5a–c and 6a–c present the measured and calculated dose profiles for each material assignment in 6 MV and 10 MV X-rays, respectively. Average dose deviations in the coil region compared to measured values across all depths are; aluminum: −1.18%, titanium: −1.33%, stainless steel: −2.51% for 6 MV X-ray and − 0.81, −1.03, −1.84% for 10 MV X-ray. Figure 7 shows the average pass rates for different material assignments. Aluminum yielded the highest pass rate, exceeding titanium by 6.4% for 6 MV X-ray and 1.8% for 10 MV X-ray. Stainless steel performed low pass rates, with pass rates of 46.5% at 6 MV X-ray and 62.0% at 10 MV X-ray.

**Fig. 8:**
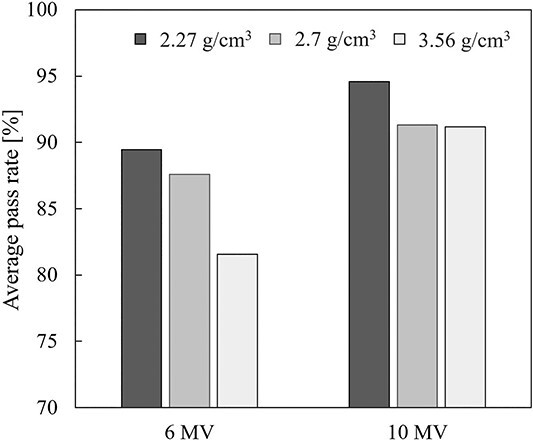
The average pass rates of each density.

Figure 8 shows the average pass rates measured with different density assignments. In 6 MV X-ray, the pass rate was highest at 2.27 g/cm^3^ with 89.5%, and the pass rate decreased with increasing density. In 10 MV X-ray, the pass rates at 2.7 and 3.5 g/cm^3^ were similar (91.3 and 91.2%, respectively), while the pass rate at 2.27 g/cm^3^ was highest at 94.6%.

**Fig. 9:**
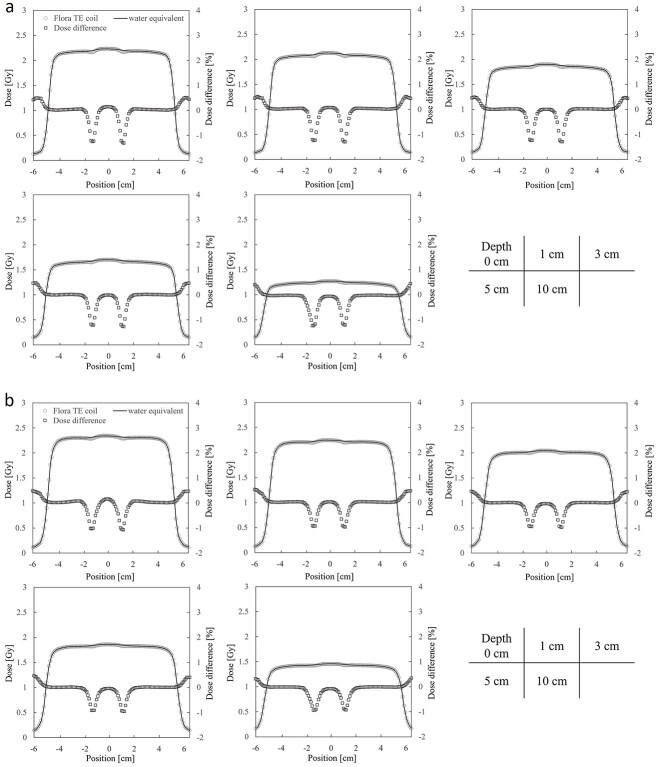
(**a**) Calculated dose profiles for 6 MV X-ray with a 2.27 g/cm^3^ aluminum coil and water equivalent in the coil. (**b**) Calculated dose profiles for 10 MV X-ray with a 2.27 g/cm^3^ aluminum coil and water equivalent in the coil.

Figure 9a and b shows the calculated dose profiles with a 2.27 g/cm^3^ aluminum coil and water equivalent in the coil. The geometrical settings and irradiation conditions were identical to those in Fig. 2 and Table 1, respectively. Across all evaluated depths, the averages and standard deviations of the coil-induced dose change were 1.29% ± 0.03 for 6 MV X-ray and 0.99% ± 0.05 for 10 MV X-ray.

### Validation of the modeling

**Fig. 10:**
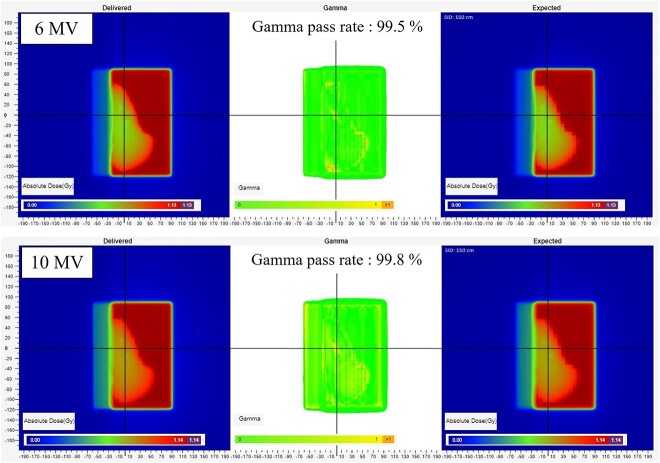
The results of the *in vivo* analysis for the modeling validation. The coil material is aluminum with a density of 2.27 g/cm^3^. The coil is present in the center of the crosshairs. The measured fluence is displayed on the left, and the planned fluence is displayed on the right. The results of the gamma analysis are shown in the center.

Following the results of the modeling procedure, the material and density assigned to the coil were aluminum and 2.27 g/cm^3^. Figure 10 shows the results of the *in vivo* analysis. There were no areas surrounding the coil where the gamma values exceeded acceptable values. The SunCHECK patient was precalibrated for absolute dose, resulting in transmitted beam images directly indicating absolute dose in the imaging plane. This enabled confirmation of agreement with both the dose distribution and absolute dose, even in the presence of the Flora TE.

## DISCUSSION

We conducted dose attenuation measurements of the RFID coil in the Flora TE and modeled the material and density for accurate PMRT planning. X-ray attenuations by the coil compared with water equivalent were 1.29% for 6 MV X-ray and 0.99% for 10 MV X-ray. For a TE with a magnetic port, 4.4% dose attenuation was observed throughout the port region under conditions similar to our measurements using 6 MV X-rays [6]. In comparison, the dose attenuation by the Flora TE coil was smaller and occurred only in some areas of the port. However, dose attenuation in the parallel direction to the coil may be greater than in the perpendicular direction. Therefore, if the attenuation is not taken into account when irradiating parallel to the coil, as in tangential PMRT, dose variations exceeding 3% may occur. To comply with the ESTRO booklet No. 7 guidance, modeling of the coil is necessary to simulate accurate dose distributions with an RTPS.

For modeling of the material and density in the coil, the most suitable assignments were aluminum and 2.27 g/cm^3^, respectively. Among the three materials compared, aluminum exhibited the closest attenuation to the actual value. However, both other metals, even when assigned their lowest possible density within the given range, showed greater attenuation than aluminum. Therefore, achieving a closer match would be impossible. Even though the coil is made of copper, stainless steel, which has the closest physical properties to copper, resulted in the largest DD. This may be due to the relationship between the actual size of the coil and the contour volume. Although the actual coil has a height and thickness of 2 mm and 2.1 mm, the defined contour encompasses a larger region due to several factors. First, it includes artifacts and a nearby air layer. Second, partial volume effects and limitations of the spatial resolution of the image in clinical use. Third, precision of ‘Segment High Density Artifacts’ function. These factors contribute to its size exceeding the actual coil dimensions and variation in CT-values observed within the contoured area. Therefore, assignment of the material according to the physical properties results in higher attenuation than that actually occurring, which leads to disagreement with the measured values. In the AcurosXB algorithm, if the region of high-density artifact is modified, dose calculation may not be started, and therefore assigning a lower attenuation value than the true value of the material seems a reasonable approach.

In our study, we employed a 10 cm × 10 cm irradiation field for modeling. Although beam quality may vary with field size and irradiation technique (e.g. volumetric modulated arc therapy, non-physical wedge, etc.) [12–14], these variations are typically smaller than those arising from nominal energy variations, which were the focus of our investigation. Consequently, the results of this study suggest that the developed model may be applicable to a broader range of irradiation methods. Based on our findings, we propose the following workflow for PMRT planning with the AcurosXB algorithm when a Flora TE is present; (1) segment the Flora TE using the ‘Segment High Density Artifacts’ function in the treatment planning CT, (2) assign aluminum with a density of 2.27 g/cm^3^ to the segmented region in the treatment planning system.

In our validation of the model, the gamma analysis of *in vivo* dosimetry did not fail, and the model seemed reasonable for clinical use. However, it is important to note that the representation of CT-values, material handling and identification of high-density areas may differ depending on the specifications of the CT scanner and the RTPS vendor.

## CONCLUSION

We evaluated the dose attenuation of the Flora TE RFID coil and identified modeling parameters for accurate PMRT planning. The coil showed dose attenuations of 1.29% for 6 MV X-rays and 0.99% in 10 MV X-rays. By modeling the coil as aluminum with a density of 2.27 g/cm^3^, acceptable calculation results could be obtained with the Eclipse AcurosXB algorithm.
